# Enhanced Fluorescence Imaging of Live Cells by Effective Cytosolic Delivery of Probes

**DOI:** 10.1371/journal.pone.0010459

**Published:** 2010-05-03

**Authors:** Marzia Massignani, Irene Canton, Tao Sun, Vanessa Hearnden, Sheila MacNeil, Adam Blanazs, Steven P. Armes, Andrew Lewis, Giuseppe Battaglia

**Affiliations:** 1 Department of Biomedical Science, University of Sheffield, Sheffield, United Kingdom; 2 Department of Engineering Materials, University of Sheffield, Sheffield, United Kingdom; 3 Department of Chemistry, University of Sheffield, Sheffield, United Kingdom; 4 Department of Oral & Maxillofacial Medicine & Surgery, School of Clinical Dentistry, University of Sheffield, Sheffield, United Kingdom; 5 Biocompatibles UK Ltd, Farnham, United Kingdom; University of Milano-Bicocca, Italy

## Abstract

**Background:**

Microscopic techniques enable real-space imaging of complex biological events and processes. They have become an essential tool to confirm and complement hypotheses made by biomedical scientists and also allow the re-examination of existing models, hence influencing future investigations. Particularly imaging live cells is crucial for an improved understanding of dynamic biological processes, however hitherto live cell imaging has been limited by the necessity to introduce probes within a cell without altering its physiological and structural integrity. We demonstrate herein that this hurdle can be overcome by effective cytosolic delivery.

**Principal Findings:**

We show the delivery within several types of mammalian cells using nanometre-sized biomimetic polymer vesicles (a.k.a. polymersomes) that offer both highly efficient cellular uptake and endolysomal escape capability without any effect on the cellular metabolic activity. Such biocompatible polymersomes can encapsulate various types of probes including cell membrane probes and nucleic acid probes as well as labelled nucleic acids, antibodies and quantum dots.

**Significance:**

We show the delivery of sufficient quantities of probes to the cytosol, allowing sustained functional imaging of live cells over time periods of days to weeks. Finally the combination of such effective staining with three-dimensional imaging by confocal laser scanning microscopy allows cell imaging in complex three-dimensional environments under both mono-culture and co-culture conditions. Thus cell migration and proliferation can be studied in models that are much closer to the *in vivo* situation.

## Introduction

Among the various microscopic techniques available, fluorescence microscopy [1], [2] (FM) has been by far the most influential. This technique allows both structural and functional information relating to cells and tissues to be obtained for routine and also more complex analysis. FM is now used in all aspects of the life sciences and has been exploited by the physical sciences as well. Over the last few decades, technological advances include total internal reflection fluorescence microscopy [3], [4], confocal laser scanning microscopy [5], [6], two-photon imaging [7], single molecule fluorescence [8], Förster energy transfer imaging [9], fluorescence lifetime imaging [10]. Most recently, new fluorescence techniques have been developed that allow nanometer resolution [11]–[13], enabling researchers to study complex biological processes in unprecedented detail. An essential aspect of FM is the fluorescence probe itself. Indeed, either a positive (i.e. functional imaging) or a negative (i.e. cytotoxicity) interaction between the probe and the biological environment dictates the correct choice of probe. New probes have been designed to target specific biological sites and/or to produce measurable signals that can be directly correlated to biological function and/or activity [14]–[16]. In addition to its spectroscopic characteristics and interaction with its biological target, the selected probe must also be introduced within cells. In particular, live cell imaging is crucial for an improved understanding of dynamic biological processes; this technique clearly requires probes that can be delivered within a cell without altering its physiological and structural integrity.

Penetration of the cell membrane depends on both the size and polarity of the probe. Small lipophilic molecules normally diffuse across the membrane quite readily, while large, polar molecules often require membrane permeabilization. However, this approach is typically either confined to one cell at time (i.e. microinjection) or compromised by cytotoxicity or other unwanted side effects (e.g. cellular stress, inflammation etc) [14]. Recently, live cell imaging has benefited tremendously from the discovery of fluorescence proteins [17]. These proteins can be designed to absorb and emit light at different wavelengths, providing biologists with a wide colour palette [18]. Genetic manipulation allows fluorescence proteins to be expressed within live cells and/or associated with specific sub-cellular compartments [18]. Although fluorescence proteins are clearly a very valuable life science tool, their use is currently limited by their genetic expression within cells. Indeed, the exact localisation and efficiency of such proteins is limited by the requirement to genetically modify the host cell. Notwithstanding the substantial advances made with both viral [19] and non-viral [20] transfection vectors, the expression of fluorescence proteins remains experimentally challenging.

Herein we propose a novel approach to introduce fluorescent probes within live cells that allows both the topological and temporal monitoring of cells in 2D monolayer and 3D environments. We have recently engineered polymer nanoparticles based on the self-assembly of block copolymers to form nanometer-sized vesicles (a.k.a. polymersomes [21]–[23]) that are rapidly taken up by many different cell types, with subsequent intracellular release of their payload [24]–[26]. We have demonstrated effective delivery of plasmid DNA to both primary cells and cell lines without altering their metabolic activity or even inducing pro-inflammatory reactions [24], [25]. We can control the polymersome uptake kinetics by fine-tuning their surface chemistry, surface topology, and dimensions [25], [26].

## Results

**Figure 1:**
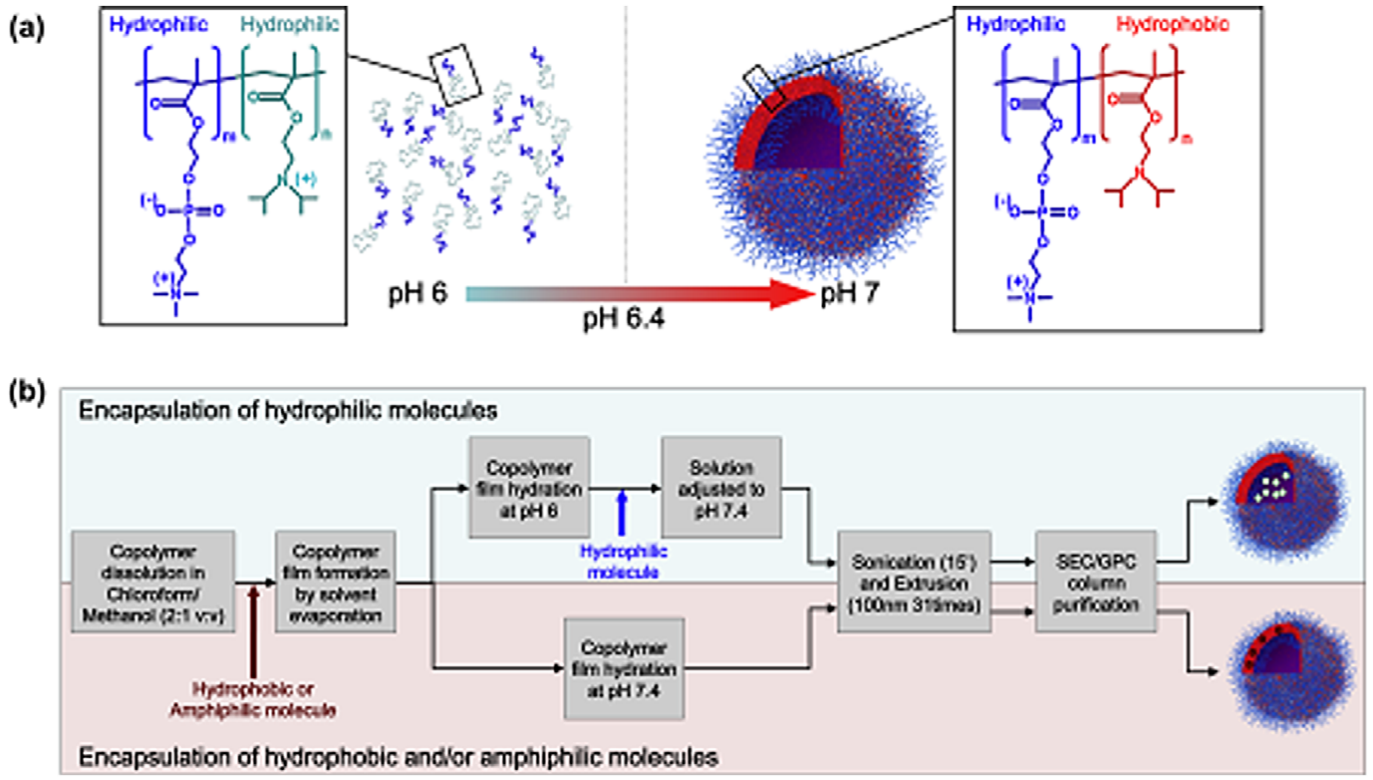
Polymersomes preparation. (a) Chemical structure and solution behaviour of the PMPC-PDPA copolymer in water at different pHs. (b) Process of encapsulations for both hydrophilic, hydrophobic and amphiphilic molecules.

Effective cytosolic delivery is achieved by the use of polymersomes comprising poly(2-(methacryloyloxy)ethyl phosphorylcholine)-poly(2-diisopropylaminoethyl methacrylate) (PMPC-PDPA) diblock copolymers (Figure 1a). The resulting polymersomes exhibit both cell affinity and pH-sensitivity (Figure 1a), which are essential for (i) cell binding and (ii) escape from the endolysomal compartments. The pH sensitivity is essential to obtain high encapsulation. Our system is able to encapsulate efficiently both hydrophilic, hydrophobic, and amphiphilic structures as schematized in Figure 1b. PMPC-PDPA copolymers were firstly dissolved in a glass vial in a 2∶1 chloroform: methanol solution. For samples encapsulating amphiphilic/hydrophobic molecules those compounds were added directly to the co-polymer chloroform: methanol solution. Subsequently, a copolymer film was formed by evaporating the solvent overnight in a vacuum oven at 37°C. The film was then rehydrated in neutral PBS for 24 hours under stirring. For encapsulating hydrophilic molecules the polymeric film was dissolved in acidic (pH 6) PBS after solvent removal. Once the film dissolved at pH 6 hydrophilic payload molecules were added. The pH of the resulting solution was raised to 7.3. Either the rehydration in neutral pH or the pH rise from 6 to 7.3 results in the formation of the polymersomes in water. The resulting polymersomes dispersions were sonicated for 15 minutes, extruded for 31 times using a 100 nm membrane and then purified by preparative gel permeation chromatography using a Sepharose 4B size exclusion column to extract the fraction containing polymersomes and remove any un-encapsulated materials.

**Figure 2:**
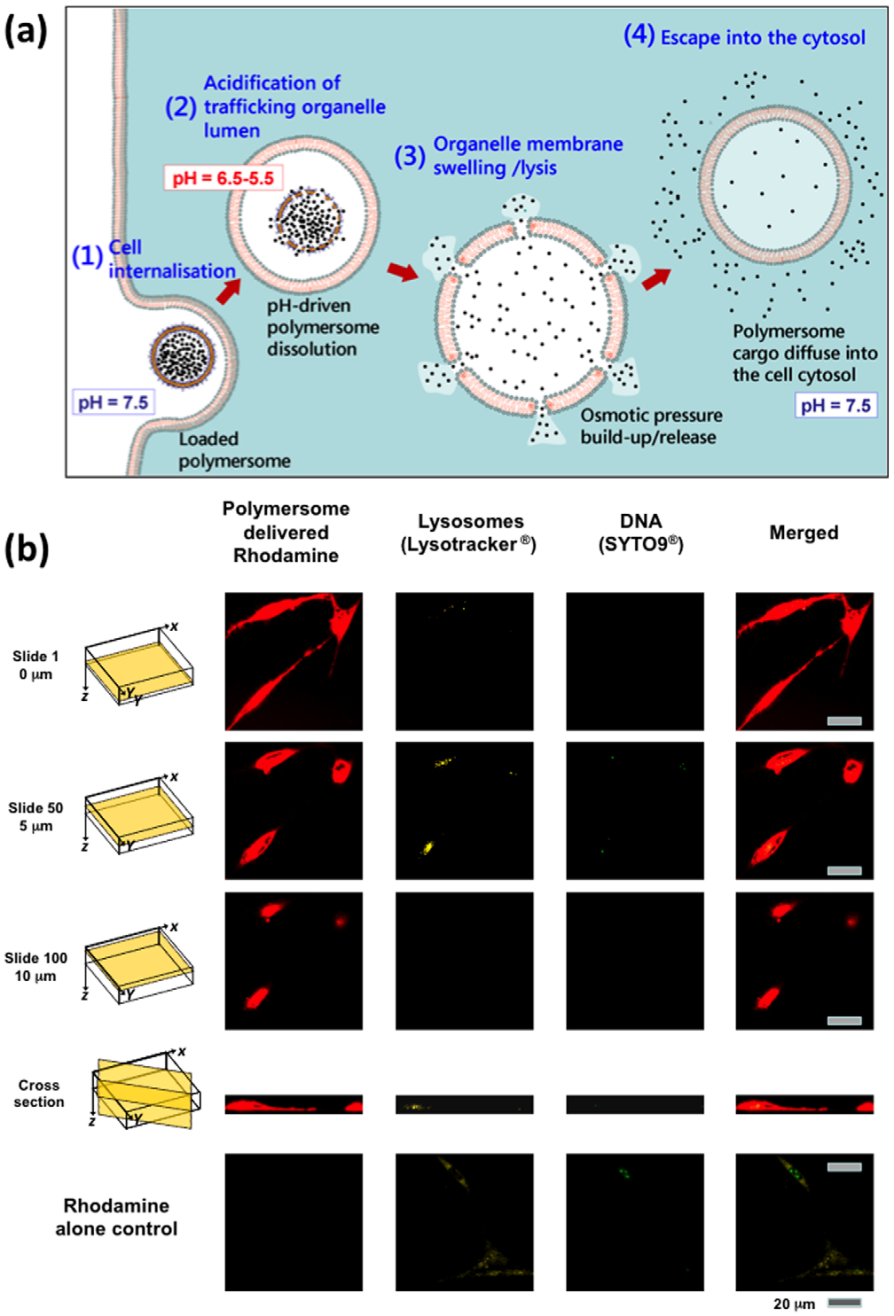
Polymersomes intracellular delivery. (a) Mechanism of polymersome-mediated cytosolic delivery. (b) Primary human dermal fibroblast (HDF) exposed to Rhodamine-loaded (red) polymersomes imaged at different focal levels (0 µm, 5 µm, 10 µm) by confocal laser scanning microscopy (40x lens, bar 0.02 mm) and compared to untreated cells. The cell lysosomes and DNA were also stained using yellow Lysotracker and green SYTO-9, respectively. Figure bar 20 µm.

The PMPC chains decorating the polymersome surface have a strong affinity for the cell membrane, thus facilitating efficient cellular uptake. Moreover, the pH-sensitive nature of the PDPA chains allows immediate polymersome dissociation once it is located within the endolysomal compartments [25], [26] (Figure 2a). This triggers a rapid build-up of osmotic pressure, leading to temporary membrane lysis and release of the polymersome payload within the cytosol. As shown in Figure 2b, primary human dermal fibroblast (HDF) cells exposed to Rhodamine-loaded polymersomes (0.005 mM) exhibit high levels of fluorescence from anywhere within their structure. Indeed, optical sections obtained by confocal laser scanning microscopy show that the dye (which otherwise would not gain entry to the cells –see control data figure 2b, dye control alone) is uniformly distributed within the cell volume. In these studies it is noteworthy that the cell lysosomes and DNA were stained with yellow Lysotracker and green SYTO-9, respectively.

**Figure 3:**
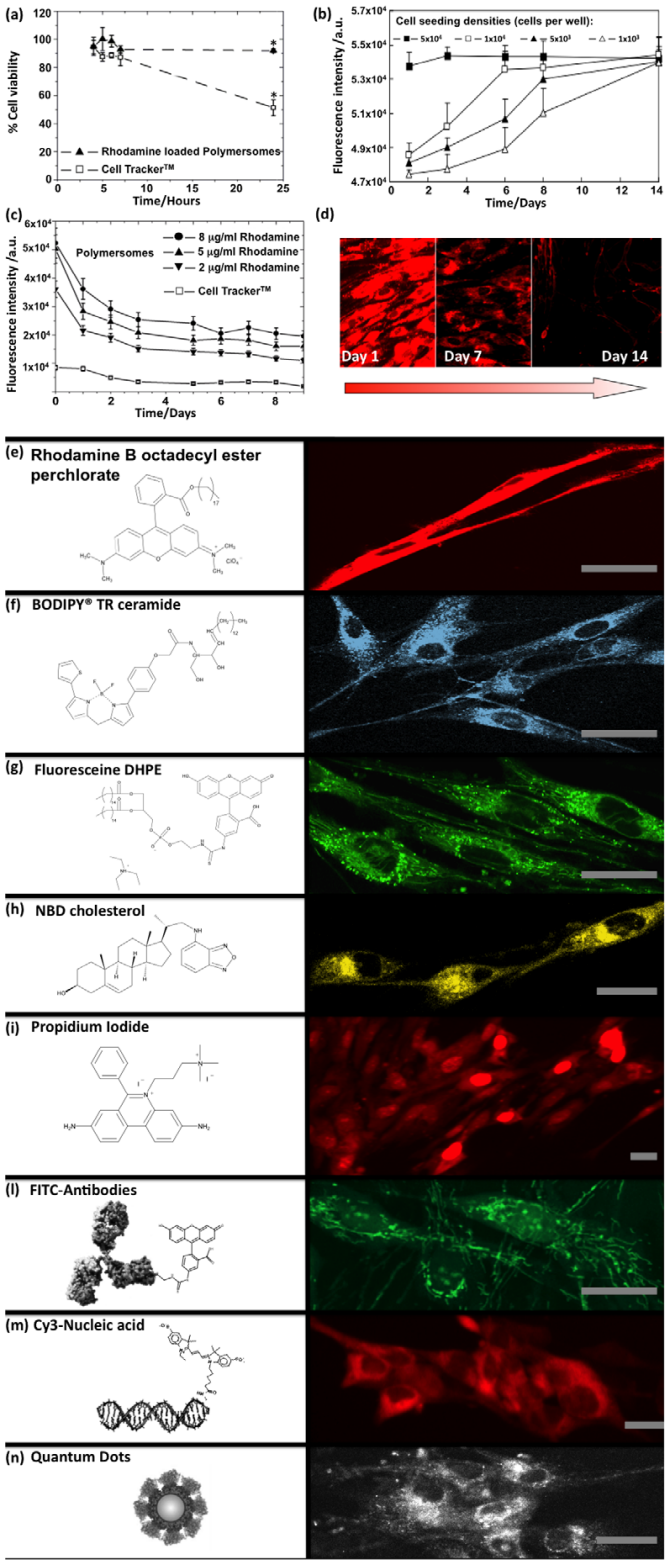
Tracking period and cytotoxicity induced by polymersome-mediated staining compared with commercial method. (**a**) Cell viability determined by MTT assay at different times for HDF cells exposed to either Rhodamine-loaded polymersomes or CellTracker (n = 3, error bar = SEM; *p<0.05). (**b**) Fluorescence intensity from HDF cells plated at different initial densities and exposed to daily dose of Rhodamine-loaded polymersomes (0.005 mM) (n = 3, error bar = SEM). (**c**) Fluorescence intensity exhibited by HDF cells after a single dose of polymersomes loaded with varying amounts of Rhodamine or CellTracker (n = 3, error bar = SEM). (**d**) Fluorescence micrographs of HDF cells recorded for the same exposure at different days after a single dose of Rhodamine-loaded polymersomes. (**e**) Fluorescence micrographs of primary HDF cells after 24 h incubation with PMPC-PDPA polymersomes loaded with membrane-staining amphiphilic Rhodamine B octadecyl ester perchlorate, (**f**) BODIPY TR ceramide, (**g**) fluorescein 1,2-dihexadecylphosphatidylethanolamine (DHPE), (**h**) labelled NBD cholesterol, (**i**) DNA staining membrane-impermeable propidium iodide, (**l**) FITC-labelled antibody anti α-tubulin, (**m**) labelled nucleic acids, (**n**) or large quantum dots. Figure bar = 0.02 mm.

We have reported previously [24]–[26] that these PMPC-PDPA polymersomes have no effect on cell viability. Provided that the fluorescent dye also has no detrimental effect, its combination with these polymersome allows fluorescence staining of live cells. This is shown in Figure 3a in which a direct comparison is made with a commercial dye, CellTracker®. This is a membrane-permeable probe modified by the addition of a thiol-reactive chloromethyl substituent that facilitates conjugation with glutathione and other intracellular peptides. These dyes are routinely used for long-term tracing applications, such as tracking cell migration during development or after transplantation [27], [28]. As shown in Figure 3a, the application of CellTracker® to live cells is limited by its toxicity, since cell viability falls to 50% after 24 hours exposure. In contrast, Rhodamine dye-loaded polymersomes, applied at the concentration tested in this work (0.005 mM), have no detrimental effect on cell viability and can be administered as multi-doses. In Figure 3b, the fluorescence intensity exhibited by HDF cells plated at different initial densities and exposed to daily doses (0.005 mM) of Rhodamine-loaded polymersomes increases with time depending on the cell density. This indicates that Rhodamine-loaded polymersomes do not affect the cell viability and indeed the extent of cell proliferation. The better performance of the polymersomes as a cell tracker agent is also shown by the enhanced fluorescence intensity over time after a single dose (figure 3c–d).

As shown in Figure 3e–3n, polymersome-mediated staining is not limited to Rhodamine B dyes; efficient encapsulation of hydrophilic, hydrophobic, and amphiphilic dyes can also be achieved [23]. These can therefore be chosen to target specific cell compartments such as the cell membranes using Rhodamine B octadecyl ester perchlorate (Figure 3e), phospholipids, BODIPY TR ceramide (Figure 3f) and Fluoresceine 1,2-dihexadecylphosphatidylethanolamine (DHPE) (Figure 3g), and labelled NBD cholesterol (Figure 3h). Similarly, using the strong nucleic acid binder, Propidium Iodide, both DNA and RNA can be highlighted (Figure 3i). One advantage of using these PMPC-PDPA polymersomes is their proven ability to encapsulate large macromolecules. Recently, we have demonstrated the encapsulation and delivery of antibodies within cells [29], [30]. The use of antibodies opens up the possibility of targeting almost any subcellular compartments for the functional imaging of live cells. This is illustrated in Figure 3l, where effective staining of live HDF cell tubulin is achieved by delivering mouse monoclonal anti-human-a-tubulin FITC-labelled IgG (anti-human-a-tubulin). Similarly, we have demonstrated the effective delivery of nucleic acids with subsequent high transfection efficiencies [24], [25], [31]. This allows the tracking of labelled nucleic acids, as shown in Figure 3m, which in principle enables the study of genetic expression, nucleic acid stability and mobility within the cytosol. Finally, we have encapsulated and delivered relatively large (about 2.6 nm) inorganic quantum dots (LumidotTM CdSe/ZnS) into cells (see Figure 3n).

**Figure 4:**
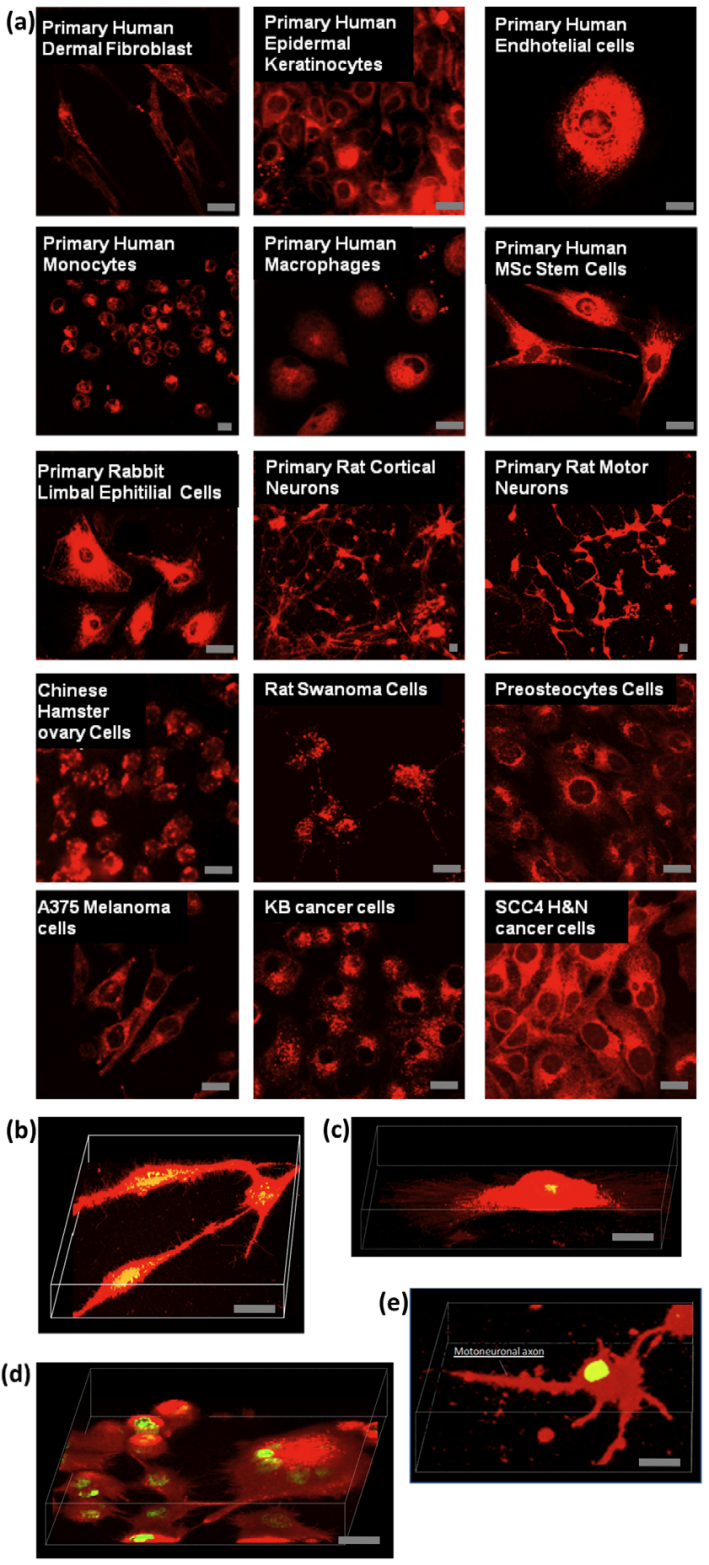
Selected examples of cells treated with Rhodamine-loaded polymersomes. (**a**) Live primary human dermal fibroblast (HDF), primary human epidermal keratinocytes (HEK), primary human endothelial cells (HE), primary human monocytes (HMC), primary human macrophages (HMP), primary human mesenchymal stem cells (HMSc), primary rabbit limbal epithelial (RLE) cells, primary rat cortical neurons (RCN), primary rat motor neurons (RMN), Chinese hamster ovary (CHO) cells, rat Shawnoma (nemap22) cell, preosteocytes (MLO-A5) cells, human melanoma (A375SM) cell, human head & neck cancer (KB) and (SCC4) cells have all been exposed to Rhodamine-loaded polymersomes and successfully stained. 3D reconstruction from confocal laser scanning optical stacks of HDF cells (**b**), HE cells (**c**), SCC4 cells (**c**), and RMN cells (**d**). Figure bar = 0.02 mm.

In summary, we have confirmed efficient cellular uptake of polymersomes using 22 different types of animal and human cells, including both primary cells and cell lines [26]. In Figure 4, selected examples of stained cells are shown, including primary human dermal fibroblast (HDF), primary human epidermal keratinocytes (HEK), primary human endothelial cells (HE), primary human monocytes (HMC), primary human macrophages (HMP), primary human mesenchymal stem cells (HMSc), primary rabbit limbal epithelial (RLE) cells, primary rat cortical neurons (RCN), primary rat motor neurons (RMN), Chinese hamster ovary (CHO) cells, rat Shawnoma (Nemap22) cell, preosteocytes (MLO-A5) cells, human melanoma A375SM cells, human head & neck cancer KB and SCC4 cells. In each case the live cells were imaged with no prior fixation. The combination of such effective fluorophore delivery with confocal laser scanning microscopy allows high quality acquisition of optical stacks and consequent 3D reconstruction. As shown in Figures 4b–e, this allows visualisation of a large number of structural details, such as the adhesion filipodia in both HDF and HE cells, the multinuclear nature of SCC4 cancer cells, and the formation of motoneuronal axon on RMN cells. Such fine details are only observed because relatively large quantities of fluorescence dye can be delivered to these cells without compromising their viability.

**Figure 5:**
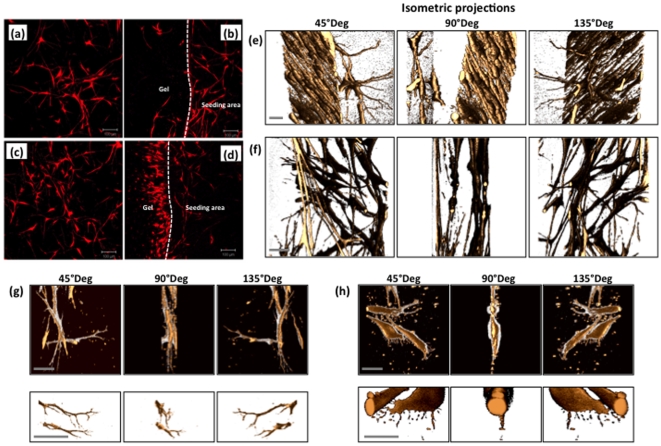
HDF cells seeded in a pre-cast fibrin clot gel stained using Rhodamine B octadecyl ester-loaded polymersomes and subsequently monitored using fluorescent microscopy. (**a**) Imaged after 7 days at the initial cell seeding area. (**b**) Imaged after 7 days at the interface between the initial cell seeding area and the pre-cast gel. (**c**) Imaged after 14 days at the initial cell seeding area. (**d**) Imaged after 14 days at the interface between the initial cell seeding area and the pre-cast gel. Figure bar = 0.1 mm. 3D volumetric reconstruction of HDF cells migrating within fibrin-clot gels imaged at the migration front areas (**e**) and at the initial cell seeding area (**f**). Details of single cells and their filipodia imaged at the seeding area (**g**) and the migration front (**h**). Figure bar = 0.01 mm.

**Figure 6:**
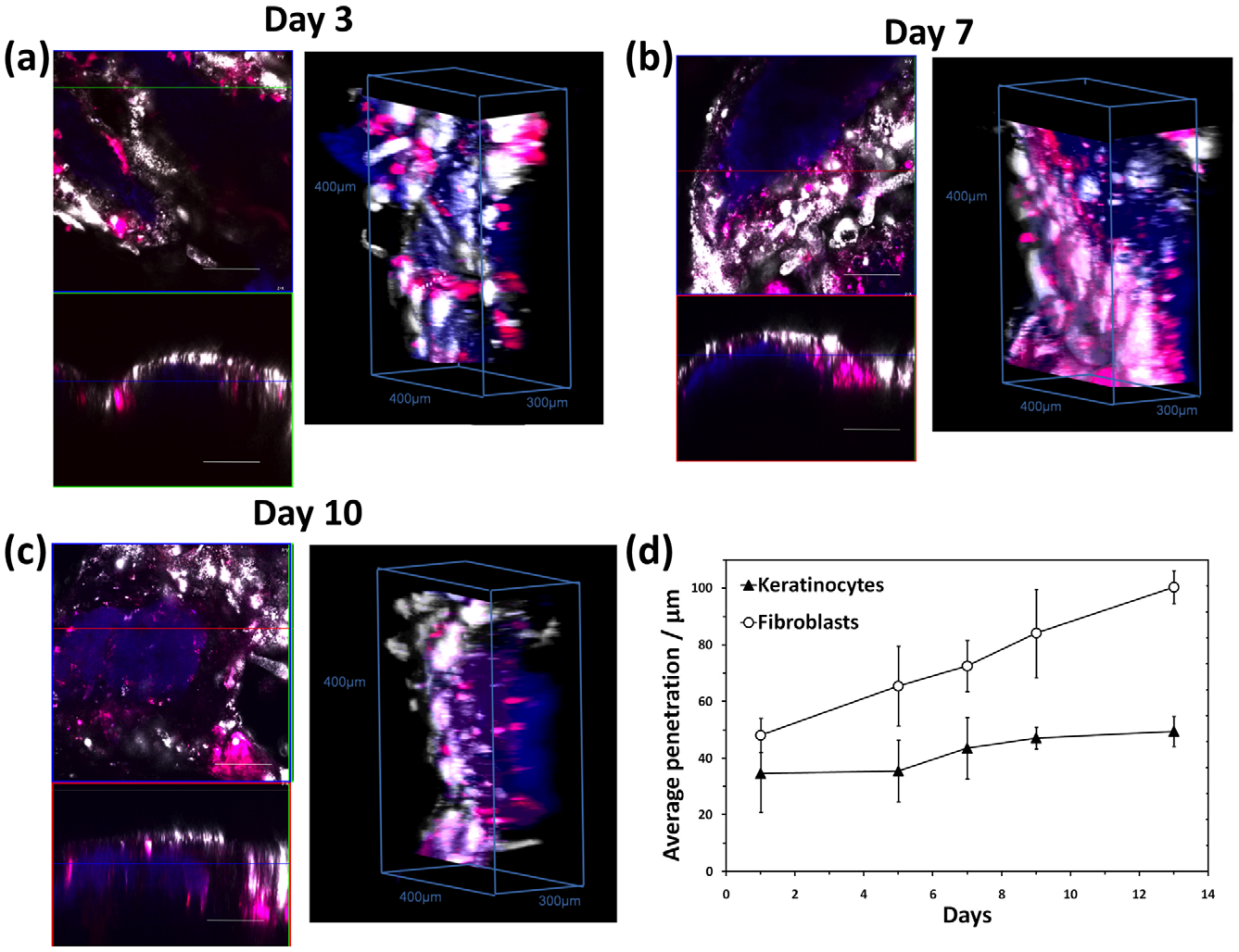
Tracking different cell types in 3D models. (**a**) Tissue-engineered human oral mucosa imaged by confocal laser scanning microscopy after 3 days, (**b**) 7 days and (**c**) 10 days. The keratinocytes (HOKs) and the fibroblasts (HOFs) that comprise the mucosa were previously stained by incubating the cells with Rhodamine B octadecyl ester- (red) and (1,1′-Dioctadecyl-3,3,3′,3′-tetramethylindodicarbocyanine perchlorate)- (white) loaded polymersomes. (**d**) The blue channel shows endogenous autofluorescence from the connective tissue component 3D cell imaging allows the extent of dye permeation to be determined over time (error bar = SEM, n = 3). Between 90 and 110 slices were acquired for each image. Slice thickness 0.003 mm.

Polymersome-mediated staining combined with confocal laser scanning microscopy has also allowed us to image cells in complex 3D environments. As shown in Figure 5a–d, HDF cells can be imaged as cultured in 3D fibrin clot gels. The nanometer-sized polymersomes penetrate rapidly into the fibrin clot. As shown in Figure 5a, cell staining was efficient enough to allow visualisation after 7 days from cell seeding, both at the original cell seeding area (Figure 5a) and also at the interface between the seeding area and the gel (Figure 5b). Facile application of the polymersomes allows the cell motility to be monitored in detail by fluorescence, as illustrated by the series of micrographs recorded for the cell seeding area (Figure 5c) and also the cell moving across the seeding area and the interface (Figure 5d). This latter experiment shows the motility front both in terms of cell number and cell morphology. Further details of the cell morphology were obtained by confocal laser scanning microscopy. In Figures 5e–h, 3D isometric projections of volume reconstructions from different focal plane micrographs show the morphology of HDF cells in different locations within a range of 100 µm at submicron spatial resolution. Such systematic analysis shows that HDF cells have different morphologies and/or alignments depending on how far they penetrated within the protein gel. Elongated and aligned HDF cells formed very regular, densely-packed multi-cellular structures both within and on the surface of the pre-cast gel between the initial cell seeding and the migration front areas (Figure 5e). On the same area, individual cells were also observed to sprout randomly from the aligned morphology to assume a more dendritic morphology (Figure 5e). This dendritic morphology was also observed for cells that were still localized in the initial cell seeding area (Figure 5f). Here HDF cells are linked by the dendrites to form a random 3D network. These dendrites were imaged at higher magnification and are shown in Figure 5g. Cells in this area seem to have very few elongated filipodia. In contrast, cells located at the migration front far from the cell seeding area are less packed and are oriented randomly toward the cell-free zone of the pre-cast fibrin gel, as shown in Figure 5h. Cells exhibited lamellipodia-like structures with many tiny filopodia-like structures (Figure 5h). Such structural details are essential to shed light on cell mobility and cell proliferation in 3D, which is much closer to the in vivo scenario. On another level of complexity, 3D fluorescence tracking can be used to assess how different cells co-operate to form complex tissues. Over the last decade, we have developed several three-dimensional tissue-engineered models of human tissue that more accurately reflect the in vivo situation [32], [33]. Such constructs offer a powerful tool to aid our understanding of how cells behave and co-operate when forming complex tissues. Herein we observe the formation of a human oral mucosa, because we have previously shown that such constructs can be conveniently imaged using confocal laser scanning microscopy by exploiting their intrinsic auto-fluorescence [34], [35]. Tissue-engineered oral mucosa were formed by seeding primary human oral fibroblast (HOF) and human oral keratinocytes (HOK) on de-epithelialised acellular dermis (DED) [36] DED is an excellent scaffold for the formation of human epithelia as these cells have the right characteristics for hosting both stromal fibroblast (i.e. collagen and fibrin) and keratinocytes (i.e. basal membrane proteins). However, although such a complex protein network exhibits auto-fluorescence over quite a wide range, we recently found that this fluorescence becomes negligible in the red (and far red) end of the spectrum (i.e. at excitation wavelengths longer than 540 nm) [35]. We therefore exposed HOF and HOK cells to polymersomes loaded with red Rhodamine B octadecyl ester and far-red (1,1′-Dioctadecyl-3,3,3′,3′-tetramethylindodicarbocyanine perchlorate) respectively. As shown by the confocal laser scanning micrograph in Figure 6 for both the x-y and x-z planes and also the 3D volumetric reconstructions, the enhanced fluorescence staining allowed tracking of the two different cells over long times. Even ten days after staining, the cells were clearly visible within the DED matrix. In addition, the multicolour imaging allows tracking of the two cell types (white and red) and their localisation respect to the protein matrix (blue). As expected, these cells behaved very differently after seeding. While the keratinocytes began to form typical colonies that gradually transformed into a thick epithelium layer, the fibroblasts started to migrate within the DED matrix and became separated from the keratinocytes. This is clearly visible after 10 days (Figure 6c), with the two cell types now almost completely separated from each other. Such behaviour can be quantified by measuring the z-average penetration over time as shown in Figure 6d. The fibroblasts migrate almost continuously during mucosa formation, while keratinocytes prefer to remain on the surface and start organizing into a thick epithelium.

## Discussion

The efficacy of fluorescence imaging of live cells can be substantially improved, both in terms of targeting and temporal staining, by exploiting pH-sensitive polymersomes to ensure effective cytosolic delivery of the fluorophore. As shown in Figure 3c, HDF cells treated with Rhodamine-loaded polymersomes are considerably more fluorescent than those treated with CellTracker™. In both cases, the dye fluorescence decays with time possibly due to due to secretion and cell proliferation. However, the enhanced delivery of the Rhodamine dye using the polymersomes allows fluorescence to be detected up to 14 days after exposure, as shown in Figure 3d. Given that the CellTracker dye and Rhodamine B have similar quantum yields and emission efficiencies [37], this enhanced performance must be due to the more effective dye delivery. We have confirmed efficient cellular uptake of polymersomes using 22 different types of animal and human cells, including both primary cells and cell lines. Polymersomes can encapsulate large quantities of hydrophilic, hydrophobic, and/or amphiphilic compounds, exhibit high affinity toward cells without causing cytotoxicity, and, after internalisation, deliver their dye payload within the cell cytosol. The combination of such versatile delivery vehicles with non-toxic fluorophores that target specific cell components has the potential for enormous impact on both cell and molecular biology. Finally, polymersome-mediated staining combined with confocal laser scanning microscopy for the first time has allowed us to study in details cells in complex 3D environments. This discovery clearly opens up promising new research avenues by allowing improved access to the majority of the biochemical processes that make up the complexity of life.

## Materials and Methods

### Materials

2-(Methacryloyloxy)ethyl phosphorylcholine (MPC; >99%) was provided by Biocompatibles UK Ltd. 2-(Diisopropylamino)ethyl methacrylate (DPA; Scientific Polymer Products, USA) was passed through the column supplied by the manufacturer to remove inhibitor. Copper(I) chloride (CuCl; 99.995%), 2,2′-bipyridine (bpy, 99%), 2-bromoisobutyl bromide, triethyl-amine, methanol and isopropanol were purchased from Sigma-Aldrich UK (Poole, Dorset, UK) and were used as received. Column chromatography grade silica gel 60 (0.063–0.200 mm) used for removal of the atomic transfer radical polymerisation (ATRP) copper catalyst was purchased from E. Merck (Darmstadt, Germany). Regenerated Cellulose (RC) dialysis tubing (Spectra Por® 6, molecular weight cut-off 3.5 kDa) was purchased from Spectrum Labs (Rancho Dominguez, CA, USA). 2-(N-Morpholino)ethyl 2-bromo-2-methylpropanoate (ME-Br) was synthesized according to a previously reported procedure [38]. Chloroform was purchased from Fisher Scientific, phosphate buffer saline (PBS) tablets from Oxoid Ltd, sepharose 4B and rhodamine octadecyl ester perchlorate from Sigma-Aldrich. DMEM media and foetal calf serum were bought from Biosera (UK) and L-glutamine, penicillin streptomycin and Amphotericin B were bought from Sigma (UK). Collagenase A was purchased from Boehringer-Mannheim (Lewes, UK). For the MTT-ESTA assay, 3-(4,5-dimethylthiazol-2-yl)-2,5 diphenyl tetrazolium bromide (MTT) was purchased from Sigma-Aldrich (UK) and hydrochloric acid from BDH AnalaR. Cell tracker® red, propidium iodide, BODIPY TR ceramide, were purchased by Invitrogen (UK).Quantum dots (Lumidot™ CdSe/ZnS 69346 nanopowder) were purchased from Sigma-Aldrich UK (Poole, Dorset, UK). PMPC25-PDPA70 diblock copolymer was synthesized by ATRP as reported else where [39]. Briefly, a round bottom flask with a magnetic stirrer bar and rubber suba seal was charged with ME-Br initiator (100 mg, 0.36 mmol), bpy ligand (111 mg, 0.71 mmol), MPC monomer (2.67 g, 8.93 mmol) and dissolved in methanol (4 ml). This solution was deoxygenated by bubbling N2 for 30 min, before the addition of Cu(I)Cl (35 mg, 0.36 mmol). The relative molar ratios of [MPC]∶[ME-Br]∶[CuCl]∶[bpy] were 25∶1∶1∶2. After 65 min, a mixture of deoxygenated DPA (5.33 g, 25 mmol) and methanol (6 ml) was injected into the flask. After 48 h the reaction solution was diluted through the addition of isopropanol and then passed through a silica column to remove the residual Cu catalyst. To remove any bpy from reaction product, the copolymer was dissolved in the minimal amount of isopropanol and dialyzed against water for 7 days, with daily changes of water. The resulting dispersed copolymer was freeze-dried and a colour-less copolymer was obtained.

### Methods

#### Production of polymersomes

Polymersomes encapsulating amphiphiles/hydrophobic molecules: 1×10–3 moles of PMPC25-PDPA70 copolymer were dissolved in a glass vial in a 2∶1 chloroform: methanol solution. For samples encapsulating rhodamine 0.05×10–3 moles of rhodamine B octadecyl ester perchlorate were added (Sigma) to the co-polymer solution. For polymersomes encapsulating 1,1′-Dioctadecyl-3,3,3′,3′-tetramethylindodicarbocyanine perchlorate (Sigma) 10 µg of this compound were added to the polymer solution. For polymersomes encapsulating BODIPY TR ceramide 0.05×10–3 moles of phospholipids was added to the polymer solution. For polymersomes encapsulating fluorescein 1,2-dihexadecylphosphatidylethanolamine 0.05×10–3 moles of phospholipids were added to the polymer solution. For polymersomes encapsulating NBD Cholesterol 0.05×10–3 moles of that molecule were added to the polymer solution. Afterwards a copolymer film was formed by evaporating the solvent for 2 hours in a vacuum oven at 37°C. The film was then rehydrated using 2 ml pH 2 PBS (100 mM). Once the film dissolved the pH was gradually increased to 7.3 in order to obtain a 0.05 mM polymer solution. The polymersomes solution was then purified by gel permeation chromatography using a sepharose 4B size exclusion column to extract the fraction containing polymersomes and removing any un-encapsulated. Polymersomes encapsulating hydrophilic molecules: 1×10–3 moles of PMPC25-PDPA70 copolymer were dissolved in a glass vial in a 2∶1 chloroform: methanol solution. Afterward a copolymer film was formed by evaporating the solvent for 2 hours in a vacuum oven at 37°C. The film was then rehydrated using 2 ml pH 2 PBS (100 mM). Once the film dissolved the pH was gradually increased. At pH 6 payloads were added (0.01 mg of propidium iodide, 0.01 mg of quantum dots (Lumidot™ CdSe/ZnS 69346 nanopowder), 0.08 mg/ml of antibodies (mouse monoclonal anti-human-a-tubulin FITC-labelled IgG), 10 ng of RNA. The pH of the resulting solution was risen to 7.3 before sonication for 15 mins using a sonicator (Sonicor Instruments Corporation). The polymersomes solution was then purified by gel permeation chromatography using a sepharose 4B size exclusion column to extract the fraction containing polymersomes and removing any un-encapsulated. In all cases the polymersomes size distribution determined by Dynamic light scattering (DLS) was 200 nm±50 nm.

#### Cell culture

Primary human dermal fibroblasts: (HDF) were isolated from skin obtained from abdominoplasty or breast reduction operations (according to local ethically approved guidelines, NHS Trust, Sheffield, UK). Primary cultures of fibroblasts were established as previously described [40]. Briefly, the epidermal layer of the skin was removed by trypsinisation and the remaining dermal layer was washed in PBS. The dermis was then minced using surgical blades and incubated in 0.5% (w/v) collagenase A at 37°C overnight in a humidified CO2 incubator. A cellular pellet was collected from the digest and cultured in DMEM (Sigma, UK) supplemented with 10% (v/v) foetal calf serum, 2 mM L-glutamine, 100 IU/ml penicillin, 100 mg/ml streptomycin and 625 ng/ml amphotericin B. Cells were sub-cultured routinely using 0.02% (w/v) EDTA and used for experimentation between passages 4 and 8. Primary human oral fibroblasts (HOF) and normal human oral keratinocytes (HOK) were isolated from oral mucosal biopsies obtained from consenting patients during dental surgeries. The biopsies were incubated overnight at 4°C in 0.1% w/v Difco trypsin solution supplemented with 100 IU/ml penicillin, 100 mg/ml streptomycin and 0.625 µg/ml amphotericin B. The epithelium was then peeled from the connective tissue component and HOKs were gently scraped from the underside of the epithelium and the top side of the connective layer using a scalpel. HOKs were cultured according to the method by Rheinwald and Green. Briefly, cells are cultured on an irradiated mouse fibroblast (i3T3) feeder layer in Green's media. Green's media is composed of Dulbecco's modified Eagle's medium (DMEM) and Hams F12 medium in a 3∶1 (v/v) ratio supplemented with 10% (v/v) FCS, 100 IU/ml penicillin, 100 mg/ml streptomycin and 625 ng/ml amphotericin B, 100 nM cholera toxin, 10 ng/ml epidermal growth factor (EGF), 0.4 µgml-1 hydrocortisone, 0.18 mM adenine, 0.005 mg/ml insulin, 0.005 mg/ml transferrin, 2 mM glutamine and 200 nM triiodothyronine. HOKs were cultured until 80% confluent and the media was replenished every 3–4 days. i3T3 cells were detached using 0.02% EDTA solution before trypsinisation of the HOKs. HOFs were isolated from the connective tissue component of the oral biopsy. The connective tissue was finely minced and incubated at 37°C in a 5% CO2 humidified incubator overnight in 10 ml of 0.5% collagenase A. The isolated HOFs were cultured in DMEM supplemented with 10% (v/v) foetal calf serum (FCS), 100 IU ml-1 penicillin, 100 mg/ml streptomycin and 625 ng/ml amphotericin B. HOKs were used up to passage 3 and HOFs between passage 3 and 8. Human squamous cell carcinoma: (FADU) whrere cultured in RPMI supplemented with 10% FCS, 2 mM L-glutamine and 50 U/ml penicillin & streptomycin. Wild type Chinese hamster ovary cells (CHO) were cultured in Ham's F12 medium containing 10% FCS, 2 mM L-glutamine, 50 U/ml penicillin, and 0.05 mg/ml streptomycin and 300 ng/ml amphotericin B. Cells were sub-cultured routinely using 0.02% (w/v) EDTA.

Macrophage and monocytes culture and extractions: Fresh isolated blood was collected into 50 ml Falcon tube containing 3.7% w/v sodium citrate. Blood solution was spun down for 20 minutes at 400 g/1400 rpm. Serum was collected to make platelet rich plasma derived serum (PRPDS). 0.026 ml of CACl2 1 mM was added to every ml of the serum and the solution was incubated at 37°C for 1 hour. Blood cells were then mixed 1∶1 with HBSS (Cambrex/BioWhittaker, Cat no. BE10-547F). Subsequentially 30 ml of blood/HBSS was overlaid onto 20 ml Ficoll-Hypaque Lymphocyte Separation Media (LSM1077 – PAA Labs, Cat no. J15-004). Blood fractions were spun for 40 minutes at the same rpm as before to be separated. At this point the Monocyte layer (approx 50 ml) was collected and dispersed in HBSS media. Then the tube was spun down for 15 minutes, and the supernatant was discarded. Finally the cell number was counted. Cells has been plate at 20×10^6^ in a 6-well plate in IMDM (Sigma, Cat no. BE12-76F) +2% human AB serum (Sigma, H4522-100 ml)+2 mM L-Glutamine. To allow mononuclear cells adhesion cells were left for 2 hours at 37°C, 5% CO_2_. Then the medium was removed and the cells were washed 4 times with HBSS. Finally cells were left in 10 ml IMDM +2% AB serum +2 mM L-Glutamine and incubated at 37°C, 5% CO_2_.

#### Cells treatment with polymersomes

Cells were seeded in standard 6 well plates at a density of 5×10^4^ cells per well and grown for two days in culture medium. Medium was fresh replenished added with 0.005 mM polymersomes encapsulating molecules. Cells were incubated for 24 hours. Treated samples were then washed 5 times with PBS and immediately visualized by live confocal fluorescent scanning microscopy. Results were compared with untreated samples. MTT-ESTA assay: All cells types were seeded in standard 24 well plates at a density of 3×10^4^ cells per well and grown for two days in culture medium. Blood cells (macrophages, monocites and red blood cells) were left and treated in suspension. Cell monolayers/suspension was treated for 24 h with fresh medium containing 0.005 mM of polymersomes. Viable cell density was then assessed using the MTT (3-(4,5-dimethylthiazol-2-yl)-2,5 diphenyl tetrazolium bromide) assay. Briefly, treated/untreated cells were washed thoroughly twice with PBS and then incubated in MTT solution (0.5 mg/ml MTT in PBS, 1 ml per well of 24 well plate or per cm2 of cultured tissue) for 45 minutes at 37°C and 95% air/5% CO_2_. Intracellular metabolic activity reduces MTT to a purple formazan salt. Subsequently, the solution was aspirated and the insoluble formazan product was solubilised by adding isopropanol (0.5 ml per well of 24 well plate or 1 ml/cm 2 cultured tissue) and incubating for 10 minutes. The optical density at 540 nm was then measured using a plate reading spectrophotometer (Dynex Technologies, MRX II). The statistical analysis performed on the data was a paired Student's t-test.

#### Cell treatment with cell tracker red

The cell tracker red lyophilized product was dissolved DMSO to a final concentration of 10 mM. The stock solution was then diluted down to a final working concentration of 0.005 mM in serum-free medium and applied to cells at 37°C for 45 minutes. Cells were then washed three times with PBS and the applied solution was replenished with full medium. The statistical analysis performed on the data was a paired Student's t-test.

#### Culture of 3D tissue engineered oral mucosa

Skin obtained from consenting donors was de-cellularised in 1 mol/L sodium chloride for at least 8 hours and washed thoroughly in PBS and cell culture medium to create de-epithelialised dermis (DED). When HOFs and HOKs were 70–80% confluent, dye containing polymersomes were added to the cell culture medium giving a final concentration of 1 mg polymer/ml cell culture media. HOFs and HOKs were incubated for 24 hours with Rhodamine B octadecyl ester perchlorate and 1,1'-Dioctadecyl-3,3,3',3'-tetramethylindodicarbocyanine perchlorate containing polymersomes respectively. Cells were then trypsinized before being added to the 3D culture as a cell suspension. DED was cut into 2 cm×2 cm squares and placed into 6 well plates submerged in Green's media. 1 mL of cell suspension containing 5×105 polymersome labelled HOKs and 5×10^5^ polymersome labelled HOFs in Green's media was added into stainless steel rings pushed onto the DED. Green's media was also added outside the ring to stop the cell solution leaking out. After 2 days half the media inside the ring was changed. On day 3 the DED with cells attached was brought to an air liquid interface using a stainless steel grid and the first image was obtained. The underside of the model was in contact with Green's media while the top was exposed to the air to encourage epithelial stratification. Models were cultured for 10 days at the air-liquid interface (ALI) and imaged using an upright Zeiss LSM 510 Meta confocal microscope. Models were washed 3X with PBS before imaging and submerged in PBS during imaging.

#### Cell seeding using fibrin clot

Fibrin clot was used to seed cells at specific areas for some of the 2D and all the 3D culture experiments. Briefly, cell suspension was prepared by mixing fibrinogen (3.5 mg/ml), thrombin (10 units/ml) and HDFs (1×10^5^ cells per ml of fibrin fibrinogen) together. Tiny drops (10–20 µm) of the cell suspension were quickly transferred to specific areas of either 2D or 3D culture systems. After incubated at 37°C for 20–40 minutes, the fibrinogen polymerised and the cells were embedded in the fibrin clot. Cell culture was subsequently carried out by submerging the cells and or the 3D scaffolds in DMEM medium. In this research, whether the HDFs were stained (with either vesicles or Cell TrackerTM) or not, the cell densities in all the cell suspensions were constant (1×10^5^ cells per ml of fibrin fibrinogen). Slight modification was made to the previously developed 3D cell culture system using fibrin clot [41]. Briefly, plasminogen-free bovine fibrinogen (3.5 mg/ml) dissolved in serum free DMEM medium was mixed thoroughly with bovine thrombin (10 IU/ml), and then quickly transferred into a silicon mould attached to 6-well tissue culture plate to cast a thin fibrin gel with the thickness of 0.2–0.5 mm. After the fibrin matrix gelled within 20–40 minutes, HDFs were seeded specifically on one end of the pre-cast gel using the cell seeding method described earlier. Cell culture was then carried out by submerging the gel and the cells in DMEM medium at 37°C, 95% air/5% CO2. During cell culture in the 3D fibrin clot scaffold, the media were changed 2–3 times a week. To monitor the performance of the culture systems and or investigate cell-cell cell-scaffold interactions at different culture time points, the 6 well plates were taken out of the incubator and image analysis of the cells was performed from the bottom of the plate using inverted phase-contrast or epifluorescent microscopy as described in the following sections. For confocal laser scanning microscopy, immersion objective lenses were used to monitor the cell cultured in 2D or 3D environments directly.

#### Microscopy

Both epifluorescence microscopy and Confocal laser scanning microscopy were used to monitor and analyse cells cultured on either tissue culture surface or 3D scaffolds after the cells were stained with CellTracker™ or polymersomes encapsuling Rhodamine B octadecyl ester perchlorate. Epifluorescent or confocal images were taken using ImageXpress™ (AXON, USA), fluorescent microcope (Leica, Germany) with a 10×magnification lens and Confocal Laser Scanning Microcope LSM 510 META (Carl Zeiss, Germany) at λ_ex_ = 543 nm, λ_em_ = 600 nm (for vesicle rhodamine/Cell TrackerTM visualization) with a 40×magnification lens. In order to locate cells in fibrin clot for optical sectioning of the cells in the scaffold, cell nuclei were also stained with 1 µM of SYTO 9 dye (Invitrogen, UK) for 10–15 minutes and washing thoroughly with either PBS or cell culture media for laser scanning confocal microcopy at λ_ex_ = 488 nm, λ_em_ = 543 nm. In situ analysis of cell density, integrity and distribution in 2D or 3D culture was conducted using image analysis software (Openlab 4.0.2, Volocity 3.0.2 and LSM510 META). Reconstructed 3D images were also produced using LSM510 META software by taking separated z-stacked image planes through different layers of the tissues, followed by volume reconstruction.
